# Influence of probiotic adjunct cultures on the characteristics of low‐fat Feta cheese

**DOI:** 10.1002/fsn3.2121

**Published:** 2021-01-19

**Authors:** Mahmoud E. Ahmed, Kaavya Rathnakumar, Nancy Awasti, Mohamed Salem Elfaruk, Ahmed R. A. Hammam

**Affiliations:** ^1^ Dairy Science Department Faculty of Agriculture Assiut University Assiut Egypt; ^2^ Dairy and Food Science Department South Dakota State University Brookings SD USA; ^3^ Lactalis American Group Nampa ID USA; ^4^ Medical Technology College Nalut University Nalut Libya

**Keywords:** adjunct cultures, buffalo milk, feta cheese, low‐fat cheese, probiotic bacteria, sensory characteristics

## Abstract

There are different methods that have been recently applied to develop a process to manufacture low‐fat Feta cheese (LFC) with acceptable flavor and texture. The objective of this study was to produce LFC from skim buffalo's milk (SBM) using *Streptococcus thermophilus* (ST) and *Lactobacillus bulgaricus* (LB) as control LFC (T1) incorporated with other probiotic adjunct cultures (PAC), such as *Lactobacillus casei* (LBC) in T2, *Bifidobacterium bifidum* (BB) in T3, and *Lactococcus lactis* subsp. *lactis* (LL) in T4. The SBM was pasteurized and inoculated with 3% of starter cultures; then, 0.4% of rennet and 3% of salt were added. After coagulation, the cheese was cut, packed, and stored at 4°C. The chemical, microbiological, and sensory characteristics of LFC were monitored during 14 days of storage. The moisture, acidity, total protein (TP), salt, and fat of LFC were approximately 75.0%, 1.0%, 17.0%, 3.0%, and 1.2%, respectively, after 14 days of storage at 4°C. The viability of PAC was high (5–7 log cfu/g) at the end of storage, which makes LFC a functional product with a valuable source of probiotic. Moreover, the adjunct cultures improved (*p* < .05) the sensory characteristics of LFC, including the texture and flavor.

## Graphical abstract

Probiotic adjunct cultures improved the characteristics and nutritional value of low‐fat Feta cheese.

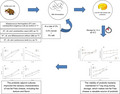

## 1 INTRODUCTION

Feta cheese is a popular soft cheese in many countries in Africa, Europe, and other regions. It was commonly manufactured from goat milk, but nowadays different types of milk, including sheep, cow, and buffalo's milk are utilized to produce Feta cheese. Traditionally, Feta cheese has been manufactured from raw milk; however, recently milk is pasteurized before being utilized in making Feta cheese due to the variations in the flavor and characteristics of cheese over the year. The natural microflora in raw milk resulted in short ripening time and intense flavor, but it can lead to unfavorable defects in the cheese characteristics or consumer health due to the pathogenic bacteria. Consequently, the typical Feta cheese is produced from pasteurized milk to assure the safety of consumers and to maintain the typical characteristics of cheese over the year. However, Feta cheese produced from pasteurized milk has various challenges because of its lack of flavor intensity as compared to Feta cheese made from raw milk (Bintsis & Robinson, 2004; Hamdy et al., 2020).

Consequently, several studies have focused on producing Feta cheese from pasteurized milk using different starter cultures, additives, different sources of milk, and changes in process conditions to stimulate the flavor and texture relative to raw Feta cheese. The typical process of Feta cheese is produced using lactic acid bacteria (LAB). The LAB in Feta cheese is typically elevated with increasing the acidity at the beginning of the ripening when it is stored at 5–7°C and then the count of LAB becomes constant up to 60 days (Bintsis & Robinson, 2004). On the other hand, mesophilic starter cultures are decreased at the prematuration of Feta cheese especially in the existence of higher salt content (6%–8%) and pH of <5.0. Therefore, thermophilic and probiotic bacteria have been used as adjunct starter cultures to enhance the flavor of Feta cheese type. The flavor of cheese is obtained during the metabolism activity of the bacteria on different components, including protein (proteolysis), lactose (fermentation), and fat (lipolysis). Several probiotic strains have been utilized recently in the manufacture of different types of cheese as adjunct starter cultures, such as *Bifidobacterium bifidum* (BB) (Peirotén et al., 2019), *Lactobacillus casei* (LBC) (Randazzo et al., 2008), and *Lactococcus lactis* subsp. *lactis* (LL) (Michaelidou et al., 2003) attributable to their metabolism activity in producing desirable flavors (Champagne et al., 2009). Furthermore, probiotic bacteria have been used to provide health benefits to the consumers when 5–7 log cfu/g exist in the product (Hamdy et al., 2020; Roy, 2001).

Although the production of low‐fat cheese (LC) has challenges because of the lack of fat which in turn has a significant effect on the flavor and texture characteristics of cheese, the sales and market of LC have increased recently due to the health problems of full‐fat cheese (FC). The FC can result in several health issues, namely elevated blood pressure, cardiovascular diseases, increased cholesterol levels, and obesity (Michaelidou et al., 2003) due to the high fat content. As a consequence, many researchers have been working on manufacturing LC by mimicking its flavor and texture characteristics to that of the FC (Bintsis & Robinson, 2004). Many modifications have been applied to the traditional process of making LC to compensate for the low level of fat, such as using different starter cultures and increasing the moisture content in cheese to make the texture softer and smoother. The most common method, which is utilized nowadays to produce high‐quality LC, is using probiotic adjunct cultures (PAC) which can enhance the flavor and texture of cheese as similar to that of FC. Those bacteria have higher metabolism to improve the proteolysis (degrading of protein) which in turn can produce flavor components similar to those in FC. The adjunct cultures are well known to improve the properties of low‐fat Feta cheese (LFC) as compared to conventional starter cultures that are used in the typical process of making Feta cheese. It has been found that using adjunct cultures, such as *LL* in making LFC with 7% fat resulted in similar flavor and texture characteristics as compared to 22% fat Feta cheese (Katsiari et al., 2002). Several studies have been performed on improving the quality of LFC made from partially skimmed bovine milk (Katsiari et al., 2002; Michaelidou et al., 2003). However, there is few studies available on improving the characteristics of LFC from skim buffalo's milk (SBM) using PAC. Therefore, the objective of this work was to develop a process to manufacture LFC from SBM using starter cultures of BB, LBC, and LL as adjunct cultures.

## 2 MATERIALS AND METHODS

### 2.1 Manufacturing of LFC

**FIGURE 1:**
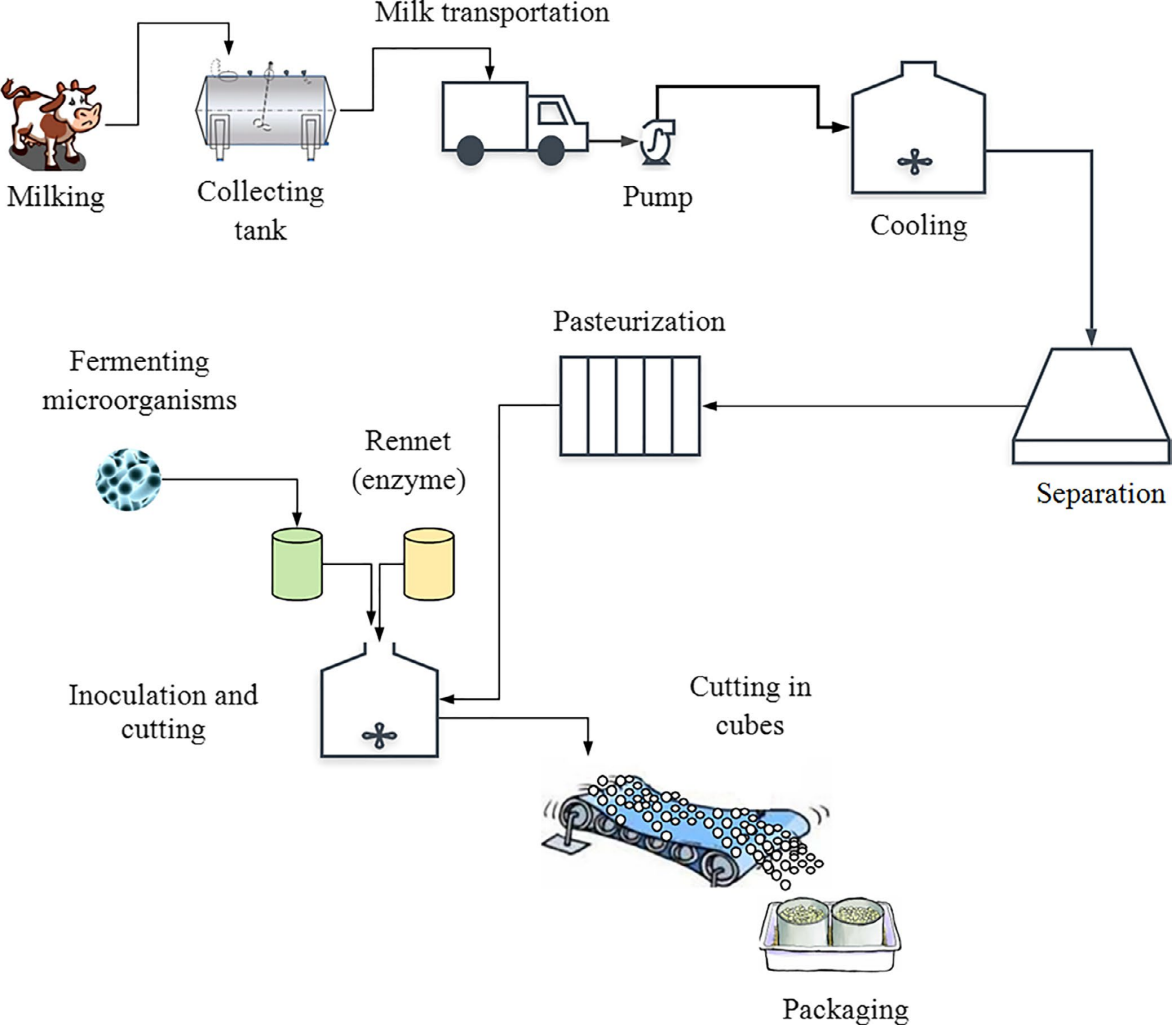
Flowchart of making low‐fat Feta cheese (LFC)

Fresh buffalo's milk (Farm of Faculty of Agriculture, Assiut University, Egypt) was separated at 4°C to produce SBM. Figure 1 shows the process of making LFC. The SBM was pasteurized at 73°C/16 s and cooled to 40°C. Aliquots of four portions of SBM were taken and added with 3% salt (sodium chloride). The first portion (T1) was typically manufactured as control LFC which was inoculated with 3% of starter cultures *Streptococcus thermophilus* (ST) and *Lactobacillus bulgaricus* (LB) (Egyptian Microbial Culture Collection: EMCC, Cairo MIRCEN, Faculty of Agriculture, Ain Shams University, Cairo, Egypt). The second portion (T2) was inoculated with 3% of starter cultures composed of ST, LB, and LBC. The third portion (T3) was inoculated with 3% of starter cultures consisted of ST, LB, and BB, while the fourth portion (T4) was inoculated with 3% of starter cultures contained ST, LB, and LL. Rennet (Chr. Hansen, Copenhagen, Denmark) was also added to all treatments at a rate of 0.4% when the acidity reached 0.2%. The fermentation time was approximately 2 hr at 40°C. Afterward, the curd was cut and subjected to drain in cheesecloth overnight at 4°C. Further, cheese was removed from the cheesecloth, made into cubes, packed in sterilized glass containers, and stored at 6 ± 2°C. Chemical, microbiological, and sensory analyses were performed on the cheese after manufacturing at 0, 3, 7, 10, and 14 days of storage. The experiment was carried out in triplicates.

### 2.2 Chemical analyses

All chemicals used for this experiment were obtained from BDH, Sigma, and Prolabo Chemicals companies. The samples of cheese were analyzed for titratable acidity, total protein (TP), fat, moisture, and salt. Titratable acidity was obtained by calculating the total lactic acid presented in the LFC (Sadler & Murphy, 2010). The fat content was measured by using Gerber method (Kleyn et al., 2001). The TP was determined using the Kjeldahl method (AOAC, 2000; method 991.20; 33.2.11) by utilizing multiple factors of 6.38. Moisture content was determined using a forced draft oven (AOAC, 2000; method 990.20; 33.2.44). The salt content was also calculated using the Mohr method (Johnson & Olson, 1985). The chemical composition was monitored at 0, 3, 7, 10, and 14 days.

### 2.3 Microbiological analyses

One gram of the cheese samples was weighted under the aseptic conditions and transferred into a sterilized jar. Subsequently, 9 ml of a sterile phosphate buffer was added and evenly mix to have the 1:10 dilution which was utilized for preparing the sequence of dilutions (Wehr & Frank, 2004). Total bacterial count (TBC) was plated in duplicate on nutrient agar medium, and enumeration was done using the standard plate count technique (Wehr & Frank, 2004). The plates were incubated at 32°C for 48–72 before microbial enumeration. *Lactobacillus* bacterial counts were determined using the MRS agar medium (De Man et al., 1960), and plates were incubated at 37°C for 48 hr under anaerobic conditions. The ST count was enumerated using the M17 agar medium (Wehr & Frank, 2004), and plates were anaerobically incubated at 40°C for 48 hr before enumeration. The BB was plated using thioglycollate medium (Brewer, 1940) and plates were incubated at 37°C for 48 hr under anaerobic conditions. The coliform count was enumerated on MacConkey broth media, and tubes were incubated at 32°C ± 1°C for 24 hr (Ashenafi, 1990). Yeast and mold counts were also enumerated (Wehr & Frank, 2004) using potato dextrose agar media, and plates were incubated at 25°C ± 1°C for 5 days. The microbiological analyses of LFC prepared using different treatments were performed at 0, 3, 7, 10, and 14 days.

### 2.4 Sensory evaluation

The sensory characteristics of LFC were evaluated according to 10–15 trained panelists from the Dairy Science Department, Assiut University. The LFC was examined as described in a previous study (Hamdy et al., 2020). Samples were evaluated for color and appearance (15 points), flavor (50 points), and body and texture (35 points) to have 100 points as a total. The mean values and their standard deviation were calculated. The organoleptic characteristics were evaluated weekly at 0, 7, and 14 days.

### 2.5 Statistical analysis

Data were analyzed by R software (R x64‐3.3.3). All data were analyzed by ANOVA using a GLM for each variable to study the effect of treatments and time (storage period) on the characteristics of LFC made with APC. Mean separation was done using the least significant difference (LSD) comparison test when significant differences were detected at *p* ≤ .05.

## 3 RESULTS AND DISCUSSION

### 3.1 Composition of cheese

**FIGURE 2:**
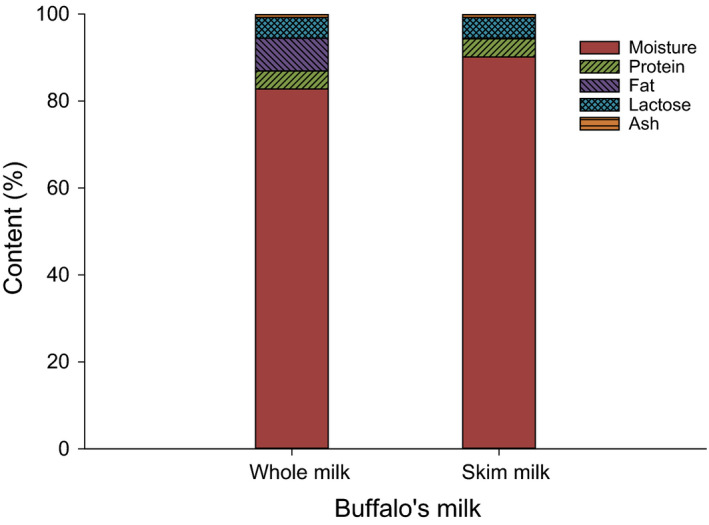
Composition of whole and skim buffalo's milk (SBM) used in making low‐fat Feta cheese (LFC)

The composition of SBM used in this study is illustrated in Figure 2. The SBM utilized to manufacture LFC had approximately 90.14%, 4.15%, 0.15%, 4.76%, and 0.8% for Moisture, TP, fat, lactose, and ash, respectively. Lactose content was calculated based on the difference in TS content.

**FIGURE 3:**
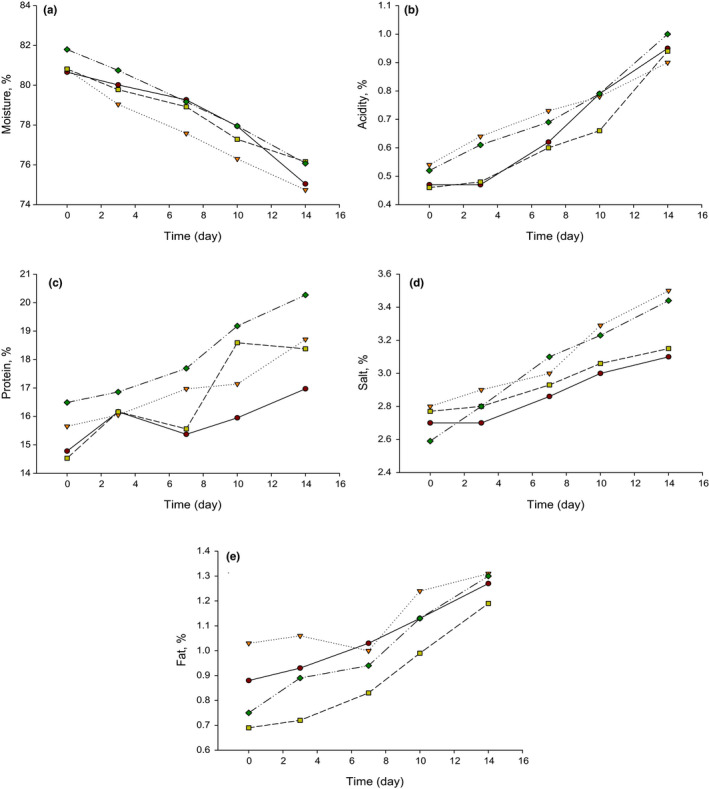
Chemical composition of low‐fat Feta cheese (LFC) made from buffalo's skim milk (SBM). T1 = (●) = Control low‐fat Feta cheese (LFC) made with *Streptococcus thermophilus* (ST) and *Lactobacillus bulgaricus* (LB); T2 = (▼) = LFC made with T1 culture plus *Lactobacillus casei* (LBC) as probiotic adjunct culture (PAC); T3 = (■) = LFC made with T1 culture plus *Bifidobacterium bifidum* (BB) as PAC; T4 = (♦) = LFC made with T1 culture plus *Lactococcus lactis* subsp. *lactis* (LL) as PAC

The mean values of moisture, acidity, protein, salt, and fat in LFC are shown in Figure 3. It was expected that the moisture content of LFC was decreased during the 14 days of storage at 4°C (Figure 3a). There was a slight difference (*p* ≤ .05) in the moisture content of all treatments, and this could be due to the slight differences in the cheese pressing times or weights utilized to drain some of the whey. It has been reported that extending pressing time led to decrease the moisture content of cheese (Everard et al., 2011). However, most of the cheese varieties had approximately 75% moisture which was high as compared to the typical moisture content in other Feta cheeses (Ahmed et al., 2005; Ayad, 2009; Carvalho et al., 2007; Verdini et al., 2004; Zalazar et al., 2002). The high moisture content in the produced cheese could compensate for the lack of fat which could result in a smooth and favorable texture (Ahmed et al., 2005). On the other hand, no significant differences (*p* ≤ .05) were detected in the acidity content of LFCs made with PAC. The acidity elevated during the 14 days of storage as was expected due to the activity of starter cultures in converting the lactose into lactic acid (Figure 3b). The acidity content increased from around 0.5% to 1% during storage at 4°C. A similar trend was reported on different types of Feta cheeses (Ayad, 2009; Bintsis & Robinson, 2004).

While the moisture content of the cheese decreased, it was expected that the TP (Figure 3c), salt (Figure 3d), and fat (Figure 3e) values were increasing during storage at 4°C. The small differences in TP, salt, and fat contents in typical LFC (T1) and PAC cheeses (T2, T3, and T4) at the beginning and end of storage could be due to the manufacturing efficiency of curd which resulted in some loss of TP, salt, and fat in the whey. The typical TP content of LFC is ranged from 14 to 20% which was similar to the TP content in this study (Ahmed et al., 2005; Katsiari et al., 2002; Michaelidou et al., 2003; Zalazar et al., 2002). Also, the salt content of the produced LFC had a similar trend to those amounts reported in other LFC and Karish cheeses (Hammam et al., 2020; Katsiari et al., 2002; Michaelidou et al., 2003). The high salt content could result in weak curd which is unfavorable. As a result, approximately 3% of salt was suitable to produce LFC with acceptable texture. Using SBM with 0.15% fat resulted in approximately 0.8% fat cheese at the beginning of the maturation and this value soared up to 1.2% after 14 days of storage. A slight increase in fat content was expected due to the decreasing in moisture content during storage.

### 3.2 Survival of starter cultures during the storage

**FIGURE 4:**
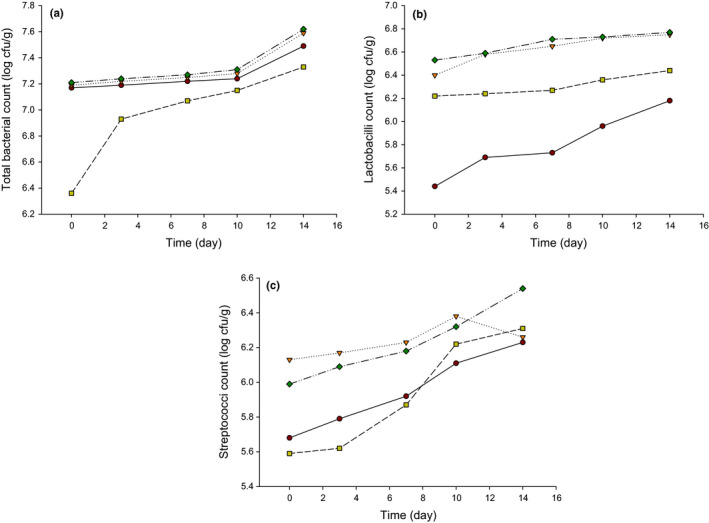
Microbiological characteristics of low‐fat Feta cheese (LFC) made from buffalo's skim milk (SBM). T1 = (●) = Control low‐fat Feta cheese (LFC) made with *Streptococcus thermophilus* (ST) and *Lactobacillus bulgaricus* (LB); T2 = (▼) = LFC made with T1 culture plus *Lactobacillus casei* (LBC) as probiotic adjunct culture (PAC); T3 = (■) = LFC made with T1 culture plus *Bifidobacterium bifidum* (BB) as PAC; T4 = (♦) = LFC made with T1 culture plus *Lactococcus lactis* subsp. *lactis* (LL) as PAC

The microbiological characteristics of LFC made from SBM are shown in Table 1 and Figure 4. The total aerobic bacterial counts (TBC) slightly increased in T1, T2, and T4 up to 10 d (Figure 4a); however, the TBC soared at 14 days of storage. The TBC in T3 showed a slight increase (*p* ≤ .05) at the beginning of ripening, and then, it elevated significantly at the end of storage. The low TBC in T3 could be due to anaerobic requirements and a low growth rate of *Bifidobacterium* species in milk (Peirotén et al., 2019). It has been found that *Bifidobacterium* grows poorly in milk and dairy products as compared to lactic acid bacteria (Peirotén et al., 2019; Roy, 2005). Table 1 exemplified the BB count in T3 which was stable during the 14 days of storage, and no significant increase in *Bifidobacterium* counts was observed. The decrease in BB counts during cheese storage is supported by a previous study (Vinderola et al., 2000) that reported a decrease in *Bifidobacterium bifidum* counts from 6.4–7.6 to 5.9–7.3 log cfu/g after 9‐week storage of Fresco cheese. Overall, TBC showed a variable trend of increase in counts during 14 days of storage. Thus, current study results are in agreement with a previous report (Effat et al., 2018), who also reported a similar increase in bacterial counts during storage of low‐salt soft cheese for 14 days.

**TABLE 1:** Mean (*n* = 3) total count of *Bifidobacterium bifidum* (log cfu/g) in low‐fat Feta cheese (LFC) during 14 d of storage

Treatments^1^	(log cfu/g)				
	0 days	3 days	7 days	10 days	14 days
T1	ND	ND	ND	ND	0.00
T2	ND	ND	ND	ND	0.00
T3	7.53	7.91	7.92	8.12	7.96
T4	ND	ND	ND	ND	0.00

^1^ ND, Not detected; T1, Control low‐fat Feta cheese (LFC) made with *Streptococcus thermophilus* (ST) and *Lactobacillus bulgaricus* (LB); T2, LFC made with T1 culture plus *Lactobacillus casei* (LBC) as probiotic adjunct culture (PAC); T3, LFC made with T1 culture plus *Bifidobacterium bifidum* (BB) as PAC; T4, LFC made with T1 culture plus *Lactococcus lactis* subsp. *lactis* (LL) as PAC.

Figure 4b and c illustrates the lactobacilli and streptococci counts, respectively, in LFC during the 14 days of storage. After comparing Figure 4a–c, it was observed that the growth of adjunct cultures was higher after 14 days of storage at 4°C. It has been exhibited that low‐fat Cheddar presented a higher amount of adjunct cultures at the end of ripening (Banks et al., 1993). The growth of streptococci in the cheese was high relative to lactobacilli. Another study reported that streptococci had a high growth rate in Cheddar cheese as compared to different strains (Champagne et al., 2009). However, both streptococci and lactobacilli existed in the range of 5–7 log cfu/g to describe the cheese as a probiotic product that is beneficial from the health aspect. As a result, both streptococci and lactobacilli viability are suitable to be utilized in producing dairy products, such as Feta cheese. The current study observed no coliform, yeast, and mold counts in the LFC during the 14 days of storage at 4°C, which represents good hygiene practice during cheese preparation.

### 3.3 Sensory properties

**FIGURE 5:**
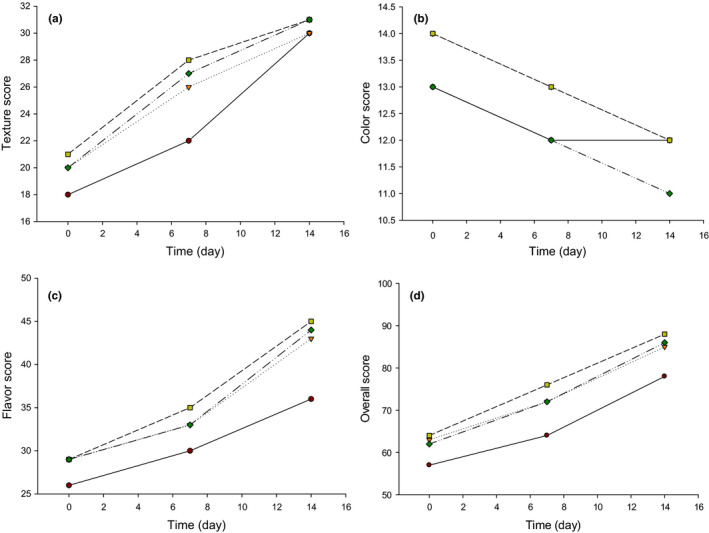
Organoleptic properties of low‐fat Feta cheese (LFC) made from buffalo's skim milk (SBM). T1 = (●) = Control low‐fat Feta cheese (LFC) made with *Streptococcus thermophilus* (ST) and *Lactobacillus bulgaricus* (LB); T2 = (▼) = LFC made with T1 culture plus *Lactobacillus casei* (LBC) as probiotic adjunct culture (PAC); T3 = (■) = LFC made with T1 culture plus *Bifidobacterium bifidum* (BB) as PAC; T4 = (♦) = LFC made with T1 culture plus *Lactococcus lactis* subsp. *lactis* (LL) as PAC

The organoleptic characteristics of LFC during the 14 days of storage at 4°C are shown in Figure 5. Implications of PAC in making LFC (T2, T3, and T4) led to improve the texture slightly (*p* ≤ .05) as compared to T1 during the 14 days of storage (Figure 5a). The high texture scores in the LFC could be due to the high moisture content in our cheese as compared to other LFC that had lower moisture content. It has been reported that the moisture content in LC could compensate for the lack of fat content by making the texture of cheese smoother (Katsiari et al., 2002). Another study has shown that the manufacture of LFC using adjunct cultures resulted in improving the body and texture of Feta cheese (Kumar et al., 2015).

Furthermore, adjunct cultures resulted in a good appearance and color of LFC except in T4. However, the color scores were reduced in all treatments during the 14 days of storage and this could be due to the loss of moisture during the ripening. A similar trend has been found in Feta cheese when it was stored for 60 days (Kumar et al., 2015). Also, other researchers noticed that the color of full‐fat and LFC was decreased during storage for 180 days (Katsiari et al., 2002) which is similar to our results. Additionally, the scores for color and appearance in Cheddar cheese made from buffalo's milk were also decreased during the ripening period (Kanawjia, 1987).

Moreover, the addition of PAC improved (*p* ≤ .05) the flavor of LFC relative to low‐fat control cheese (T1) (Figure 5c). The low flavor scores (26–29) were found at the beginning of storage due to the milk flavor. The flavor of LFC was improved during storage at 4°C for 14 days. This could be due to the higher soluble nitrogen and free amino acids produced from the activity of adjunct cultures during the ripening of LFC. It has been reported that LFC (7% fat) did not show any differences with full‐fat Feta cheese with 22% fat when adjunct cultures were utilized (Katsiari et al., 2002). A correlative trend was found in another study when Feta cheese was manufactured using adjunct cultures (Kumar et al., 2015). Another study reported that adjunct cultures improved low‐fat Cheddar cheese (Banks et al., 1993). Different studies found that the flavor of Cheddar cheese made from buffalo's milk enhanced when *LBC* was utilized as adjunct cultures (Kanawjia & Singh, 1991) which is similar to our results. Adjunct cultures have increased the overall quality of LFC as compared to control LFC (Figure 5d).

## 4 CONCLUSION

Feta cheese is a soft cheese type, and it is well‐known in many countries. Nowadays, there are different methods and trials have conducted to develop LFC with acceptable flavor and texture. This work provides insights on the characteristics of LFC made from SBM using PAC during the storage at 4°C for 14 days. The viability of PAC was high (5–7 log cfu/g) at the end of the storage period which makes the cheese valuable as a functional product. Also, adjunct cultures improved the flavor and texture of LFC during storage for 14 days. Adjunct cultures have promising applications in manufacture of cheese and other dairy products.

## Data Availability

Research data are not shared.
